# Deletion of succinic semialdehyde dehydrogenase sad and chromosomal expression of phosphoenolpyruvate carboxylase as metabolic requirements for improved production of 2,4-dihydroxybutyric acid via malyl-P pathway using *E. coli*

**DOI:** 10.3389/fbioe.2025.1589489

**Published:** 2025-05-12

**Authors:** T. A. Stefanie Nguyen, Ceren Alkim, Nadine Ihle, Thomas Walther, Cláudio J. R. Frazão

**Affiliations:** ^1^ Chair of Bioprocess Engineering, Institute of Natural Materials Technology, TU Dresden, Dresden, Germany; ^2^ Toulouse Biotechnology Institute, UMR INSA-CNRS5504, UMR INSA-INRAE 792, Toulouse, France

**Keywords:** strain engineering, synthetic metabolic pathway, 2,4-dihydroxybutyric acid, malyl phosphate pathway, succinic semialdehyde dehydrogenase, phosphoenolpyruvate carboxylase, *Escherichia coli*

## Abstract

The fermentative production of the functional precursor 2,4-dihydroxybutyrate (DHB) enables sustainable synthesis of the methionine analogue hydroxy-4-(methylthio) butyrate, which is currently still produced from fossil fuels. In this work, we aimed to optimize the aerobic production of DHB from glucose through the synthetic malyl phosphate (MalP) pathway, which comprises the conversion of the natural TCA cycle intermediate malate into MalP and the subsequent reactions to yield malate semialdehyde (MalSA) and finally DHB. We first implemented the synthetic pathway in an engineered *Escherichia coli* strain previously reported to over-produce malate through the oxidative TCA cycle. However, DHB was only detected in trace amounts, while acetate and malate were secreted in high quantities. Subsequent construction of strains producing malate, but negligible amounts of acetate, revealed that an increased supply of malate alone is not sufficient for improved production of DHB. Instead, we discovered metabolic inefficiencies in the DHB pathway as we found that deleting the endogenous succinate semialdehyde dehydrogenase Sad, whose natural substrate is structurally similar to MalSA, strongly improved performance of the DHB pathway. Specifically, with the single knock-out of *sad* we could achieve a 3-fold increase in DHB production with a yield of 0.15 mol mol^-1^ compared to the wildtype host in shake flask experiments. With additional chromosomal expression of the mutant *ppc*_*K620S*_ gene encoding the malate-insensitive phosphoenolpyruvate carboxylase under control of a weak constitutive promoter, we achieved a DHB yield of 0.22 mol mol^-1^, which corresponds to 17% of the maximal yield under aerobic conditions.

## 1 Introduction

Currently, 19 out of the 20 proteinogenic amino acids are produced via biochemical processes from renewable carbohydrates (Leuchtenberger et al., 2005; Wendisch, 2020). A notable exception is methionine, which is mainly used as feed supplement in poultry diets, and to this day still is exclusively produced from petrol at 1.5 million tons per year (François, 2023). Thus far, the biological production of L-methionine is not competitive with chemical processes. This is in part because the incorporation of sulfur into methionine is metabolically costly. Another point is that racemic mixtures of (D/L)-methionine or its 2-hydroxy-analogue (D/L)-2-hydroxy-4-(methylthio)-butyrate (HMTB), both of which can be efficiently chemically synthesized, have the same nutritional value as the L-form of the amino acid (Mitsuhashi, 2014).

**FIGURE 1:**
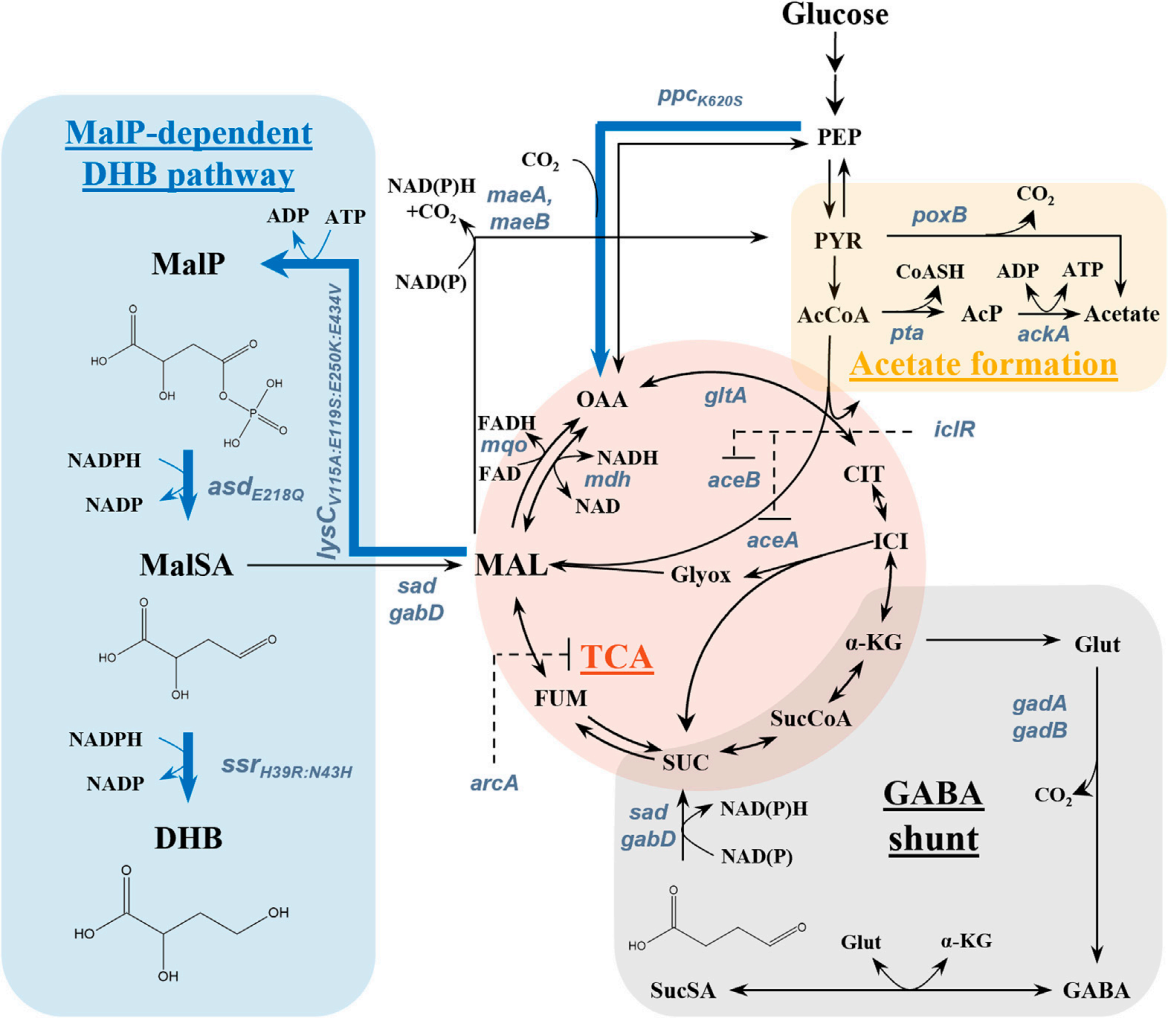
Aerobic synthesis of 2,4-dihydroxybutyric acid (DHB) from glucose via the malyl-phosphate (MalP) and relevant metabolic pathways. The synthetic DHB pathway consists of the genes *lysC*_*V115A:E119S:E250K:E434V*_ from *Escherichia coli*, *asd*_*E218Q*_ from *Bacillus subtilis* and *ssr*_*H39R:N43H*_ from *Metallosphaera sedula*. Targeted genes for strain optimization are depicted in bluish grey. Blue bold arrows show overexpressed enzymes, and dashed black lines indicate transcriptional repression by the glyoxylate shunt repressor IclR. Abbreviations: AcCoA–Acetyl-CoA; AcP–Acetyl phosphate; *α*-KG–*α*-Ketoglutarate; ASP–Aspartate; CIT–Citrate; FUM–Fumarate; GABA–*γ*-aminobutyric acid; Glut–Glutamate; ICI–Isocitrate; DHB – 2,4-Dihydroxybutyrate; MAL–Malate; MalP–Malyl phosphate; MalSA–Malate semialdehyde; OAA–Oxaloacetate; PEP–Phosphoenolpyruvate; PYR–Pyruvate; SUC–Succinate; SucCoA–Succinyl-CoA; SucSA–Succinate semialdehyde; TCA–Tricarboxylic acid cycle.

Aiming at addressing these challenges, a two-stage process was previously proposed by Walther et al. (2017) wherein the functionalized precursor 2,4-dihydroxybutyrate (DHB) is first produced from sugar by fermentation, and then converted into HMTB by established chemistry (Deck et al., 2008). This strategy enables a theoretical maximum yield of 1.33 mol mol^-1^ HTMB from glucose under aerobic conditions (Wagner et al., 2023), thus providing an attractive means to improve the economics of HMTB production from renewable resources. However, DHB is not known to occur as a natural metabolite in microbial cells (Shinka et al., 2002). Previous works focused therefore on the development of synthetic pathways enabling the direct biosynthesis of DHB from renewable sugar, and which are named after the characteristic intermediates malyl phosphate (MalP) (Walther et al., 2017), homoserine (Walther et al., 2018), and malyl-CoA (Walther et al., 2015). These pathways have different theoretical DHB yields on glucose and are different in their co-factor demand. Out of the three pathways, the homoserine pathway, in which homoserine is deaminated to 2-oxo-4-hydroxybutyrate (OHB) followed by the reduction to DHB, is the most developed production route in terms of titer (22.0 g L^-1^) (Table 1). It is of note however that those pathways are fully compatible with the use of alternative substrates which may be (bio-)chemically sourced from CO_2_ (e.g. methanol, ethylene glycol, ethanol) or plastic waste (e.g. ethylene glycol) (Vogt and Weckhuysen, 2024; Cotton et al., 2020; Gavala et al., 2021; Xia et al., 2023; Magalhães et al., 2021; Frazão et al., 2023). In the DHB pathway via MalP, the dicarboxylic acid malate serves as the link between natural and synthetic metabolism. The route proceeds through the non-natural intermediates MalP and malate semialdehyde (MalSA) by chaining the malate kinase, MalP reductase and MalSA reductase synthetic enzymatic activities (Figure 1). Upon the expression of required activities in wild-type *Escherichia coli*, DHB production starting from glucose was achieved at low-to-moderate titers (Walther et al., 2017).

**TABLE 1:** Comparison of theoretical and applied process performance of DHB producing pathways from glucose derived from different characteristic intermediates.

Intermediate	Theoretical yield [mol mol^-1^]	Co-factors	Relevant conditions	Titer [g L^-1^]	Yield [mol mol^-1^]	Ref.
Mal-P derived from malate	1.5	1 ATP 2 NAD(P)H	Plasmid-based expression of malate-insensitive phosphoenolpyruvate carboxylase (*Ec.ppc*_*K620S*_), malate kinase (*Ec*.*lysC*_*V115A:E119S:E250K:E434V*_), malate semialdehyde dehydrogenase (*Bs.asd*_*E218Q*_), malate semialdehyde reductase (*Ms*.*ssr*_*H39R:N43H*_) in wildtype *E. coli* MG1655 Shake flask cultivation (24 h). M9 medium containing 20 g L^-1^ glucose	1.8	0.15	Walther et al. (2017)
			Plasmid-based expression of malate kinase (*Ec*.*lysC*_*V115A:E119S:E250K:E434V*_), malate semialdehyde dehydrogenase (*Bs.asd*_*E218Q*_), malate semialdehyde reductase (*Ms*.*ssr*_*H39R:N43H*_) in *E. coli* MG1655 *∆sad proA-ppc*_*K620S*_ Shake flask cultivation (24 h). M9 medium containing 20 g L^-1^ glucose	3.0	0.22	This study
OHB derived from homoserine	1.5	1 NAD(P)H *α*-KG	Plasmid-based expression of homoserine dehydrogenase (*Ec.thrA*_*S345F*_), homoserine transaminase (*Ec.aspC*), OHB reductase (*Ll.ldhA*_*Q85C*_) in *E. coli* MG1655 *ΔadhE ΔldhA ΔthrB ΔmetA* Fed-batch cultivation (48 h) in 2 L fermenter in mineral medium	5.3	0.15	Walther et al. (2018)
			Plasmid-based expression of homoserine transaminase (*Ec.aspC*), OHB reductase (*Ec.mdh*_*I12V:R81A:M85Q:D86S:G179D*_) in *E. coli* BW25113 *ΔmetA ΔlysA ΔthrB ΔldhA ΔrhtA:: P*_*trc*_ *-tdcC ΔgalR::P*_*cpA1*_ *-zglf ΔptsG::P*_*119*_ *-glk ΔpflB ΔyjiP1::P*_*tac*_ -thrA* ΔyjiP2::P_*tac*_ *-asd ΔgdhA* Fed-batch cultivation (68 h) in 5 L fermenter in mineral medium	22.0	0.15	Zhang et al. (2025)
			Plasmid-based expression of malate-insensitive phosphoenolpyruvate carboxylase (*Ec.ppc*_*K620S*_), homoserine dehydrogenase (*Ec.thrA*_*S345F*_), homoserine transaminase (E*c.alaC*_*A142P:Y275D*_), OHB reductase (*Ec.mdh*_*I12V:R81A:M85Q:D86S:G179D:D34G:I35R*_) in *E. coli* MG1655 *ΔthrB ΔmetA ΔldhA pntAB* ^*proD*^ Shake flask cultivation (24 h). M9 medium containing 20 g L^-1^ glucose	2.4	0.25	Ihle et al. (2025)
Malyl-CoA derived from glyoxylate	1	2 NAD(P)H	Plasmid-based expression of malyl-CoA lyase (*Me.mcl*), malyl-CoA reductase (*St.mcr/Pg.sucD*), malate semialdehyde reductase (*Ms*.*ssr*_*H39R:N43H*_) Shake flask cultivation (24 h). M9 medium containing 20 g L^-1^ glucose	0.06	∼0.004	Walther et al. (2015)

According to the basic principles of metabolic engineering, improved performance can be achieved by increasing the intracellular availability of pathway precursor(s), in this case malate. The biosynthesis of malate in *E. coli* has been extensively researched both under aerobic and anaerobic conditions (Zhang et al., 2011; Trichez et al., 2018; Jiang et al., 2020, 2023). But as the MalP pathway requires two moieties of NADPH for the formation of DHB from malate, it is better suited for the biosynthesis of DHB under aerobic conditions (Walther et al., 2017). Thus, in preliminary work, we transferred the MalP pathway to *E. coli* chassis strains previously engineered for malate overproduction under aerobic conditions via the glyoxylate shunt (Walther et al., 2017; Trichez et al., 2018). But surprisingly, DHB yields dropped drastically. Instead, high amounts of both malate and acetate accumulated in the culture broth. In this study, we investigated the metabolic requirements for increasing the performance of the MalP pathway. We show that the deletion of all malate dehydrogenases is detrimental for DHB production and that in fact, the over-production of malate and deletion of malic enzymes is not a requirement for achieving improved DHB yields. Instead, we found the sole deletion of the promiscuous succinate semialdehyde (SucSA) dehydrogenase Sad, which we hypothesize that counter-productively converts MalSA to malate, as the key to improve DHB yield and titers. Further improvements were achieved by adjusting the chromosomal expression levels of a malate-insensitive phosphoenolpyruvate (PEP) carboxylase mutant, resulting in strains producing up to 3.0 g L^-1^ DHB at a yield of 0.22 mol mol^-1^.

## 2 Materials and methods

### 2.1 Reagents and chemicals

Unless stated otherwise, chemicals and solvents were purchased from Sigma-Aldrich (Darmstadt, Germany). Restriction endonucleases, T4 DNA ligase, Q5 DNA polymerase and DpnI enzyme were purchased from NEB (Frankfurt am Main, Germany). Kits for plasmid DNA isolation, gel DNA extraction and PCR clean-up were purchased from NEB and used according to the instructions of the manufacturer. Oligonucleotides were synthesized by Sigma-Aldrich. DNA Sanger sequencing was carried out by Genewiz (Leipzig, Germany).

### 2.2 Media

Lysogeny Broth (LB) medium (10 g L^-1^ tryptone, 5 g L^-1^ yeast extract and 10 g L^-1^ NaCl) was used for cloning procedures, strain maintenance, and cell recovery from glycerol stocks (30% v/v) kept at −80°C. LB agar plates were prepared by supplementing LB medium with 15 g L^-1^ agar-agar. M9 mineral medium was used for the biosynthesis of DHB (Walther et al., 2017). One liter of M9 medium contains: 20 g glucose, 18 g Na_2_HPO_4_·12H_2_O, 3 g KH_2_PO_4_, 0.5 g NaCl, 2 g NH_4_Cl, 0.5 g MgSO_4_·7H_2_O, 0.015 g CaCl_2_·2H_2_O, 0.010 g FeCl_3_, 0.012 g thiamine HCl and 1 ml of trace element solution (containing per liter: 0.04 g Na_2_EDTA·2H2O, 0.18 g CoCl_2_·6H2O, ZnSO_4_·7H2O, 0.04 g Na_2_MoO_4_·2H_2_O, 0.01 g H_3_BO_3_, 0.12 g MnSO_4_·H2O, 0.12 g CuCl_2_·H_2_O). The medium was buffered with 100 mM 3-(N-morpholino)-propanesulfonic acid (MOPS) that was adjusted to pH 7 with KOH. Whenever required, antibiotics were added to the media at standard concentrations (carbenicillin, 100 mg L^-1^; chloramphenicol, 35 mg L^-1^; kanamycin sulfate, 50 mg L^-1^).

### 2.3 Strain construction

All strains used and constructed in this study are listed in Table 2.

**TABLE 2:** *Escherichia coli* strains used in this study.

Strain	Genotype	Reference/Origin
NEB^®^ 5-alpha	*E. coli fhuA2 Δ(argF-lacZ)U169 phoA glnV44 Φ80Δ (lacZ)M15 gyrA96 recA1 relA1 endA1 thi-1* hsdR17	NEB™
MG1655	*F* ^*−*^ *λ* ^*-*^ *ilvG- rfb-50 rph-1*	ATCC
Mal-op	MG1655 *Δmdh Δmqo ΔmaeA ΔmaeB ΔiclR ΔarcA*	Trichez et al. (2018)
	MG1655 *Δmdh*	Trichez et al. (2018)
	MG1655 *Δmqo*	Trichez et al. (2018)
	MG1655 *Δmdh Δmqo*	Prof. J. M. François
	MG1655 *Δmdh Δmqo ΔmaeA ΔmaeB*	Prof. J. M. François
	MG1655 *Δmdh Δmqo ΔmaeA ΔmaeB ΔiclR*	Prof. J. M. François
	MG1655 *Δmdh Δmqo ΔmaeA ΔmaeB ΔarcA ΔackA-pta*	Prof. J. M. François
	MG1655 *Δmdh Δmqo ΔmaeA ΔmaeB ΔpoxB*	Trichez et al. (2018)
	MG1655 *Δmqo ΔmaeA*	Prof. J. M. François
	MG1655 *Δmqo ΔmaeB*	Prof. J. M. François
	MG1655 *Δmqo ΔmaeA ΔmaeB*	Prof. J. M. François
	MG1655 *Δmqo ΔmaeA ΔiclR*	Prof. J. M. François
	MG1655 *Δmqo ΔmaeA ΔmaeB ΔiclR*	Prof. J. M. François
	MG1655 *Δsad*	This study
	MG1655 *Δmdh Δmqo ΔmaeA ΔmaeB Δsad*	This study
	MG1655 *Δmqo ΔiclR ΔmaeA Δsad*	This study
	MG1655 *Δmqo ΔmaeA ΔmaeB ΔiclR Δsad*	This study
	MG1655 *Δsad ΔgabD*	Prof. J. M. François
	MG1655 *ΔgadA*	Prof. J. M. François
	MG1655 *ΔgadA ΔgadB*	Prof. J. M. François
	MG1655 *ΔgadA ΔgadB Δsad*	Prof. J. M. François
	MG1655 *ΔgadA ΔgadB Δsad ΔgabD*	This study
	MG1655 kan-*proA-ppc*_*K620S*_	Prof. J. M. François
	MG1655 kan-*proB-ppc*_*K620S*_	Prof. J. M. François
	MG1655 kan-*proC-ppc*_*K620S*_	Prof. J. M. François
	MG1655 kan-*proD-ppc*_*K620S*_	Prof. J. M. François
	MG1655 *Δsad* kan-*proA-ppc*_*K620S*_	This study
	MG1655 *Δmdh Δmqo ΔmaeA ΔmaeB Δsad* kan-*proA-ppc*_*K620S*_	This study
	MG1655 *Δmqo ΔmaeAB ΔiclR Δsad* kan-*proA-ppc*_*K620S*_	This study

*E. coli* K-12 substr. MG1655 (ATCC 47076) served as basis for strain construction (Table 2). Chromosomal deletions were introduced by phage transduction with the bacteriophage P1vir (Lennox, 1955), and phage lysates were created from single knock-out mutants of the Keio collection (Baba et al., 2006). To enable subsequent genetic modifications, the kanamycin resistance cassette was removed by expressing flippase (FLP) recombinase activity encoded from the pCP20 plasmid (Cherepanov and Wackernagel, 1995). Strains in which the native *ppc* operon was replaced by a FRT-kan-FRT cassette followed by the malate-insensitive *ppc*_*K620S*_ variant from *E. coli* (Yano and Izui, 1997) under control of constitutive promoters proA to proD (Davis et al., 2011) were a kind gift from Prof. J. M. François from Toulouse Biotechnology Institute, INSA Toulouse, France. The synthetic operons were then transferred to malate-producer strains by phage transduction. Chromosomal modifications were confirmed by diagnostic PCR using primers flanking the loci of interest (Supplementary Table S3), and in the case of the synthetic *ppc* operon further supported by DNA sequencing of the region encompassing the integrated promoter and gene.

### 2.4 Plasmid construction

All plasmids constructed and used for *in vivo* DHB synthesis are listed in Table 3.

**TABLE 3:** Plasmids used in this study.

Plasmid	Relevant characteristics	Reference/Origin
pCP20	*pSC101* origin, Amp^R^, Cm^R^, Flp	Cherepanov and Wackernagel (1995)
pZS13	*pSC101* origin, Amp^R^, P_A1lacO-1_ promoter	Expressys
pZA33	*p15A* origin, Cm^R^, P_A1lacO-1_ promoter	Expressys
pACT3w-ppc_K620S_	pACT3 derivative (*pACYC184* origin, Cm^R^, *tac* promoter) harboring *Ec.ppc*_*K620S*_ with weak RBS	Trichez et al. (2018)
pACT3w-ppc_K620S_-gltA_R164L_	pACT3w-ppc_K620S_ derivative harboring *Ec.gltA*_*R164L*_	Trichez et al. (2018)
pZA33-DHBop-ppc_K620S_	pZA33 derivative harboring *Ec.ppc*_*K620S*_ and the DHB operon (Walther et al., 2017) (*Ec.lysC*_*V115A:E119S:E250K:E434V*_ *; Bs.asd*_*E218Q*_ *; Ms.ssr*_*H39R:N43H*_)	Prof. J. M. François
pZA33-DHBop	pZA33 derivative harboring the DHB operon	Prof. J. M. François
pZS15-gltA_R164L_	pZS13 derivative harboring *Ec.gltA*_*R164L*_ under control of *tac* promoter	This study

#### 2.4.1 pZS15-gltA_R164L_

The 1,550-bp region including the pTAC promoter followed downstream by the *gltA*_*R164L*_ gene from *E. coli* MG1655 was amplified by PCR from the pACT3-gltA_R164L_ plasmid (Trichez et al., 2018) with the primers XhoI-pTAC-gltA_fwd and HindIII-gltA_rev (Supplementary Table S3). The purified PCR product and the plasmid backbone pZS13 (Expressys, Germany) were digested with the restriction enzymes XhoI and HindIII, gel purified and ligated. The ligation product was then transformed into chemically competent *E. coli* NEB5-α cells (NEB), and clones selected on carbenicillin-containing LB agar plates. Diagnostic PCR/restriction analysis and subsequent DNA sequencing was performed to verify plasmid correctness. Plasmids were then transformed in target *E. coli* strains using standard protocols (Sambrook et al., 1989).

### 2.5 Shake flask cultivation

All cell cultivations were carried out in an orbital shaker set at 37°C and a shaking frequency of 220 rpm (Ecotron, Infors, Switzerland). A first pre-culture was performed in a 15 mL screw cap tube containing 3 mL of LB and inoculated with a single colony. After 8 h of incubation, 0.5 mL of the pre-culture were transferred to a second pre-culture carried out in a 50 mL screw cap tube containing 10 mL of M9 mineral medium that was cultivated overnight. Then, cells were harvested by centrifugation (5 min at 6,000 g, room temperature) and used to inoculate a 250 mL baffled shake flask containing 25 mL of M9 mineral medium at an initial optical density at 600 nm (OD_600_) of 0.2. When an OD_600_ equal to 0.6 was reached, the expression of pathway genes was induced by the addition of isopropyl-β-D-thiogalactopyranoside (IPTG) at a final concentration of 1 mM. Cell growth was monitored by measuring optical densities using a GENESYS 150 UV-Vis spectrophotometer (ThermoFisher Scientific, Waltham, MA, USA).

### 2.6 Analyses of extracellular metabolites

All samples were centrifuged (2 min at 16,000 g) and the culture supernatants were kept at −20°C until analysis. Extracellular metabolites in the supernatants were analyzed by high-performance liquid chromatography (HPLC) using a Dionex UltiMate 3000 system controlled by the Chromeleon software (Thermo Scientific, USA). All samples were syringe-filtered (pore size, 0.2 µm) and the vials were loaded into the autosampler (Dionex, Sunnyvale, USA) set at 6°C. A sample volume of 20 µL was injected into the cation-exchange column Rezex™ RoA-organic acid H^+^ 8% (300 × 7.8 mm, Phenomenex, USA) preceded by a SecurityGuard™ guard cartridge (Carbo H^+^, 4 × 3 mm, Phenomenex, USA). Separation of the analytes was achieved using 0.5 mM H_2_SO_4_ as the mobile phase and keeping flow rate constant at 0.5 mL min^-1^. The temperature of the column oven was kept constant at 80°C and the samples were analyzed on a RI detector (ERC RefractoMax 520, 45°C, Knauer, Germany) for glucose, DHB, acetate, fumarate, succinate, lactate and ethanol. The UV/Vis detector was set at 210 nm (Dionex, Sunnyvale, USA) and used to analyze malate, pyruvate and formate.

The presence/absence of 4-hydroxybutyrate production was further investigated for *sad* deleted mutants by LC/MS using our previously described method (Frazão et al., 2023). Briefly, liquid chromatography (Vanquish, ThermoFisher Scientific) was performed with the Rezex™ RoA-organic acid H^+^ 8% column with a SecurityGuard™ guard cartridge held at 80°C. A mobile phase of 0.1% formic acid was used with a constant flow rate of 0.4 mL min^-1^. The samples were kept at 6°C in the autosampler and the injection volume was 20 µL. Mass spectrometry was conducted with the Thermo Scientific™ Q Exactive™ Focus device (ThermoFisher Scientific), controlled by Xcalibur software (version 2.1, ThermoFisher Scientific).

## 3 Results

### 3.1 DHB production strongly decreases in strains optimized for the aerobic biosynthesis of malate

**FIGURE 2:**
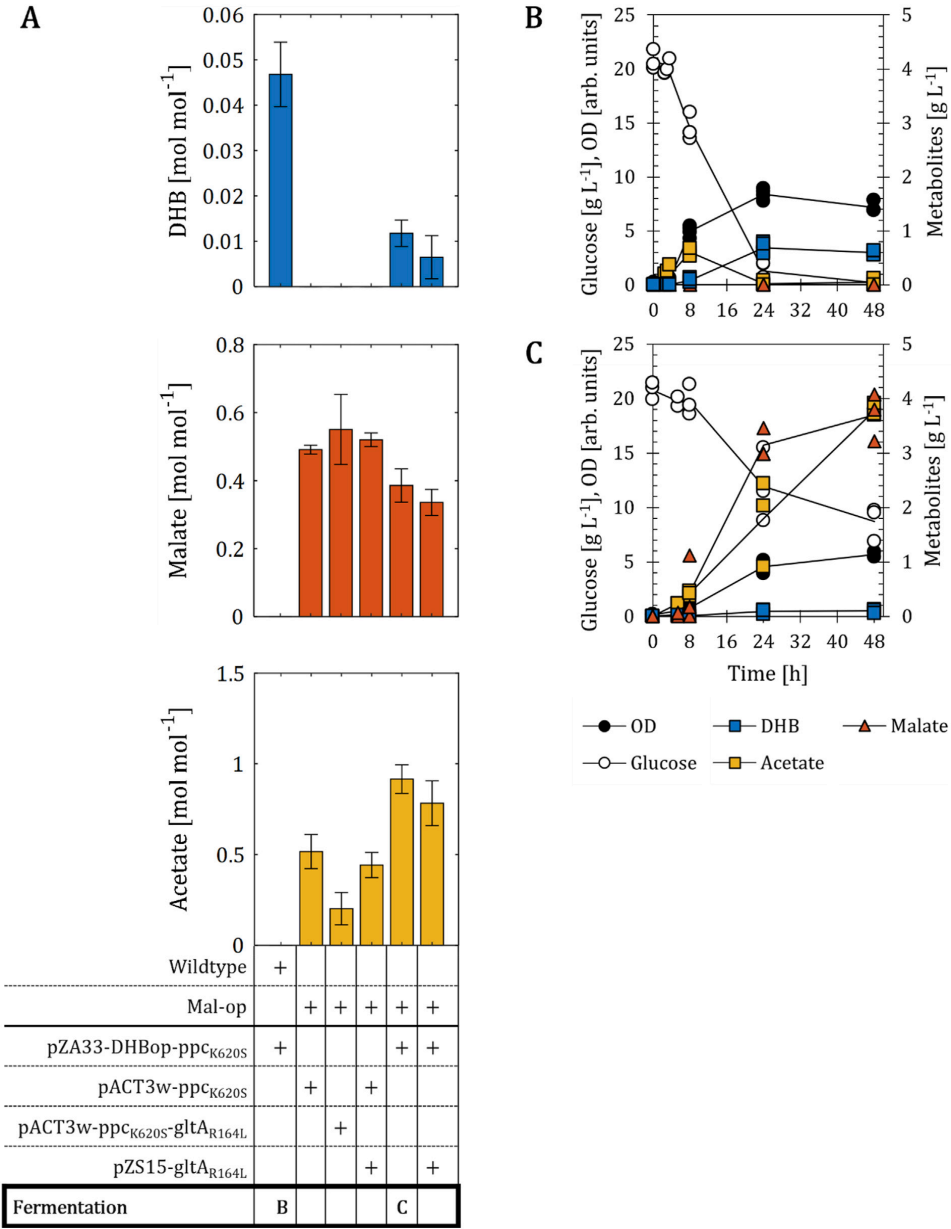
DHB production for *E. coli* mutants engineered towards increased availability of malate. **(A)** DHB, malate and acetate yields of malate optimized (Mal-op) strains expressing different production plasmids after 48 h of cell cultivation. Fermentation profile of wildtype **(B)** and Mal-op **(C)** strains expressing the plasmids pZA33-DHBop-ppc_K620S_. All strains are derived from *E. coli* MG1655. The Mal-op strain (*Δmdh Δmqo ΔmaeA ΔmaeB ΔiclR ΔarcA*) served as malate-producer host strain. Cell cultivation was performed in 250 mL baffled shake flasks (37°C, 220 rpm) with 25 mL M9 mineral medium. IPTG (1 mM) was added at mid-exponential phase to induce the expression of pathway genes. In **(A)**, results correspond to the mean of at least two biological replicates is shown and error bars represent the standard deviation of the mean. In **(B,C)**, individual data points are shown as colored dots. Lines are drawn to the mean values (see Supplementary Table S1).

The biosynthesis of DHB in *E. coli* via the MalP pathway requires the expression of genes encoding malate kinase (*lysC*_*V115A:E119S:E250K:E434V*_ from *E. coli*), MalP reductase (*asd*_*E218Q*_ from *Bacillus subtilis*), and MalSA reductase (*ssr*_*H39R:N43H*_ from *Metallosphaera sedula*) activities (Figure 1). The additional expression of the anaplerotic, malate-insensitive PEP carboxylase Ppc_K620S_ from *E. coli* has previously been found to be essential in reaching improved DHB titers under aerobic conditions (Walther et al., 2017). Therefore, we first employed the medium-copy pZA33 vector bearing the four activities under control of the IPTG-inducible promoter P_A1lacO-1_. The resulting plasmid pZA33-DHBop-ppc_K620S_ was then transformed into *E. coli* MG1655. All strain cultivations were carried out in baffled shake flasks containing mineral medium supplemented with 20 g L^-1^ glucose, and IPTG (1 mM) was added at mid-exponential phase to induce the expression of genes composing the DHB pathway. After 48 h of incubation, plasmid-bearing cells produced DHB at a yield of 0.05 mol mol^-1^, which corresponds to 3% of the maximum theoretical yield (Figure 2A). Taking into account that in this experiment no malate was detected in the culture supernatant and previous works report that wildtype cells produce negligible amounts of malate (Kövilein et al., 2020), we decided to investigate whether the biosynthesis of DHB via the MalP pathway may benefit from an increased supply of the precursor malate.

Trichez et al. (2018) previously investigated the metabolic requirements for the aerobic production of malate using *E. coli*. In that work, the malate-producing strain with the highest yield was devoid of genes encoding soluble and membrane-associated malate dehydrogenases (*mdh* and *mqo*, respectively), malic enzymes (*maeA, maeB*), the redox-regulator ArcA and the glyoxylate shunt repressor IclR (Figure 1). When this host strain (herein termed Mal-op) was equipped with a medium-copy plasmid expressing Ppc_K620S_, malate was produced at a yield of 0.49 mol mol^-1^ (Figure 2A). Acetate was the major by-product and found at a yield of 0.52 mol mol^-1^. The additional expression of the NADH-insensitive citrate synthase GltA_R164L_ (pACT3w-ppc_K620S_-gltA_R164L_), previously reported to be beneficial for malate production (Stokell et al., 2003; Trichez et al., 2018), did not change malate production significantly, even though the acetate yield could be lowered to 0.20 mol mol^-1^. Next, we transformed the Mal-op host strain with the plasmid pZA33-DHBop-ppc_K620S_. This strain produced malate at a yield (0.39 mol mol^-1^) lower than those achieved with strains devoid of DHB production plasmid, but the acetate yield increased to 0.92 mol mol^-1^ (Figure 2A). Surprisingly, the DHB yield dropped dramatically by more than 6-fold when compared to use of wildtype cells harboring the DHB production plasmid. The additional expression of GltA_R164L_ from the low-copy pZS15 plasmid did not improve DHB or malate yields and did not reduce acetate production significantly compared to the strain bearing only the pZA33-DHBop-ppc_K620S_ plasmid (Figure 2A). Likewise, the pZS15-gltA_R164L_ plasmid did not impact malate or acetate production significantly in the Mal-op strain expressing the pACT3w-ppc_K620S_ plasmid.

We further investigated the fermentation profile of the wildtype and Mal-op strains bearing the DHB producer plasmid (Figures 2B,C). In the case of the wildtype as host of the DHB pathway (Figure 2B), small amounts of acetate were produced at the early stages of cell cultivation (<0.7 g L^-1^) with no significant production of DHB. Between 8 and 24 h of cultivation however, acetate was almost entirely re-consumed. After 48 h of cultivation, 0.6 g L^-1^ DHB were produced. Throughout the cultivation malate did not accumulate in the culture broth. In the case of Mal-op as background (Figure 2C), cells produced high amounts of malate and acetate (up to 3.7 and 3.9 g L^-1^, respectively). Neither of the two fermentation products was re-assimilated during cell cultivation. Only small amounts of DHB (0.1 g L^-1^) were produced.

Therefore, we concluded that a strong supply of malate and acetate employing the Mal-op strain background has an antagonistic effect on the biosynthesis of DHB. In the following sections, we investigate the metabolic requirements for improving the performance of the DHB synthetic pathway.

### 3.2 The simultaneous inactivation of two malate dehydrogenases abolishes DHB production and causes overproduction of acetate

**FIGURE 3:**
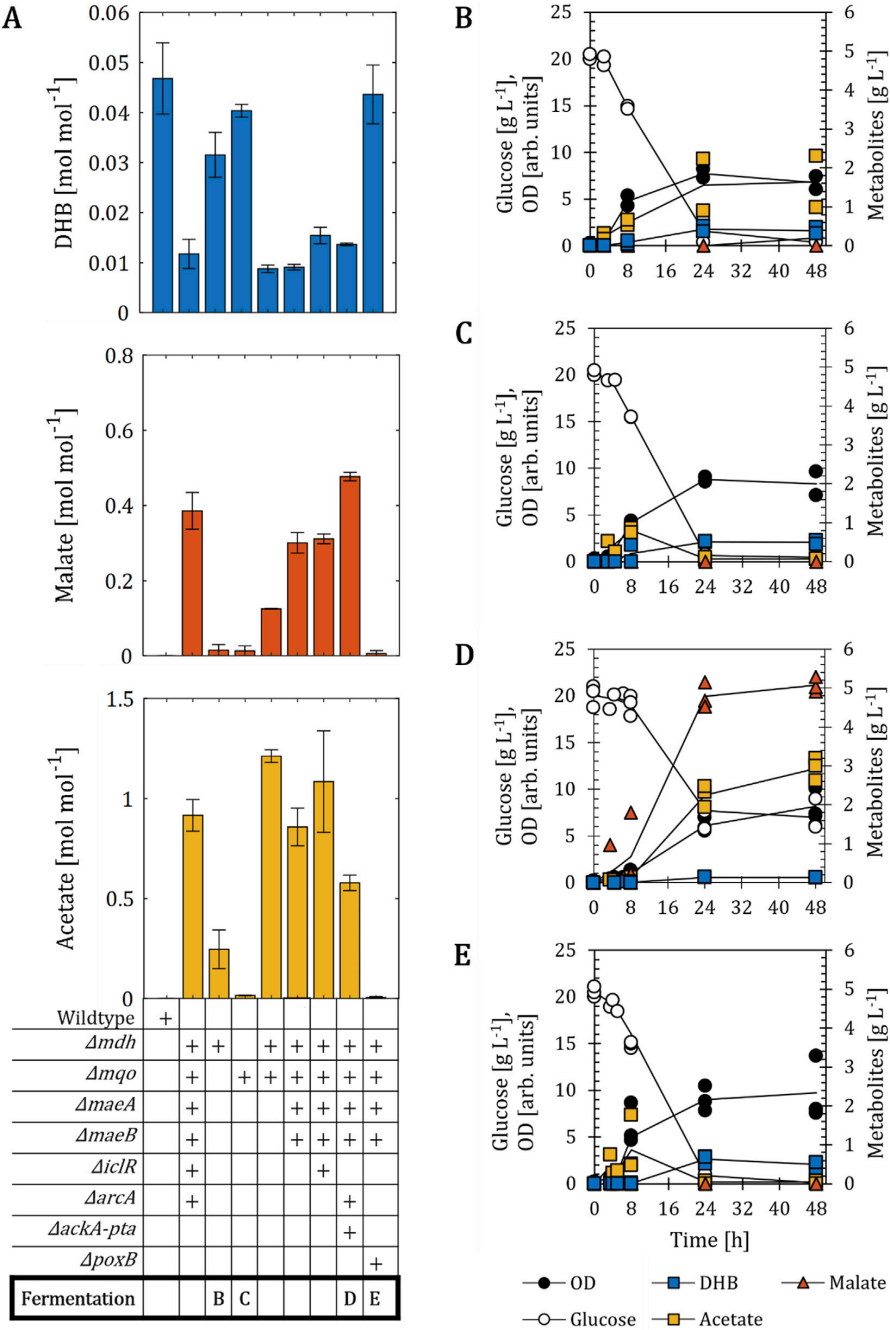
DHB production for (E) *coli* mutants devoid of both malate dehydrogenases. **(A)** DHB, malate and acetate yields for *E. coli* mutants expressing the plasmid pZA33-DHBop-ppc_K620S_ after 48 h of cultivation. Deletion (∆) of target genes is marked with a (+) sign. The *E. coli* strain *Δmdh Δmqo ΔmaeA ΔmaeB ΔiclR ΔarcA* corresponds to the Mal-op background. Fermentation profile of strains *∆mdh* **(B)**, *∆mqo* **(C)**, *Δmdh Δmqo ΔmaeA ΔmaeB ΔarcA ΔackA-pta* **(D)** *and Δmdh Δmqo ΔmaeA ΔmaeB ΔpoxB* **(E)**. All strains are derived from *E. coli* MG1655. Cell cultivation was performed in 250 mL baffled shake flasks (37°C, 220 rpm) with 25 mL M9 mineral medium. IPTG (1 mM) was added at mid-exponential phase to induce the expression of pathway genes. In **(A)**, results correspond to the mean of at least two biological replicates is shown and error bars represent the standard deviation of the mean. In **(B–E)**, individual data points are shown as colored dots. Lines are drawn to the mean values (see Supplementary Table S1).

The Mal-op strain background nearly abolished the biosynthesis of DHB. Thus, we set out to investigate the impact of the introduced genetic modifications alone or in combination on the performance of the DHB pathway (Figure 3). All strains were transformed with the plasmid pZA33-DHBop-ppc_K620S_ and DHB, malate and acetate yields after 48 h of cultivation were calculated. We first assessed the performance of *E. coli* strains devoid of malate dehydrogenase-encoding genes (*mdh*, *mqo*). The single mutants *Δmdh* and *Δmqo* produced DHB at yields (0.03 and 0.04 mol mol^-1^, respectively) lower, yet comparable to those obtained with the wildtype host strain (0.05 mol mol^-1^) (Figures 3A–C). Both mutants secreted malate to the extracellular medium at yields (0.02 and 0.01 mol mol^-1^ for *Δmdh* and *Δmqo*, respectively) much lower than those achieved with the Mal-op host strain bearing the DHB pathway. Next, we investigated DHB production from the double mutant *E. coli Δmdh Δmqo* host strain. Here, the DHB yield dropped dramatically to 0.01 mol mol^-1^ and increased amounts of malate and acetate were found to accumulate in the extracellular medium (0.13 and 1.21 mol mol^-1^, respectively). To prevent the decarboxylation of malate into pyruvate, genes encoding malic enzymes were inactivated yielding the quadruple mutant *Δmdh Δmqo ΔmaeA ΔmaeB*. This strain produced more malate and less acetate (0.3 mol mol^-1^ and 0.86 mol mol^-1^, respectively) than the strain *Δmdh Δmqo*. No increase in DHB yield was achieved with this setting when compared to the double mutant *Δmdh Δmqo*. The further inactivation of IclR (resulting in the quintuple mutant *Δmdh Δmqo ΔmaeA ΔmaeB ΔiclR*) could increase the DHB production up to 0.02 mol mol^-1^ with a small reduction of malate formation. But still, the formation of acetate could not be significantly lowered.

Taking into account that all the tested strains secreting high yields of malate have in common the production of acetate at even higher yields, we next decided to inactivate acetate-yielding pathways from the quadruple mutant *Δmdh Δmqo ΔmaeA ΔmaeB* producer strains (Figure 3A). However, the strain further deleted for the *ackA-pta* pathway produced acetate at yield of 1.08 mol mol^-1^, accompanied by a malate production of only 0.06 mol mol^-1^ (Supplementary Table S2). No DHB production was found to occur in this setting. The additional inactivation of the repressor ArcA, yielding the strain *Δmdh Δmqo ΔmaeA ΔmaeB ΔackA-pta ΔarcA*, resulted in a lower acetate yield (0.56 mol mol^-1^). While more malate was secreted (0.48 mol mol^-1^) by this strain, the DHB yield remained comparable to that observed for the quadruple mutant *Δmdh Δmqo ΔmaeA ΔmaeB* (Figures 3A,D). On the other hand, the inactivation of pyruvate oxidase activity (encoded by *poxB*), which is responsible for the conversion of pyruvate into acetate and CO_2_, restored DHB production to a titer (0.04 mol mol^-1^) comparable to that obtained with the wildtype background. The highest concentration of acetate (0.9 g L^-1^) was achieved after 8 h of cultivation, and the compound was subsequently re-assimilated (Figure 3E), which was comparable to the fermentation profile of the wildtype host strain (Figure 2B). However, only minimal amounts of malate were detected (0.1 g L^-1^) and fumarate as well as succinate were hardly produced (0.01 mol mol^-1^, Supplementary Table S2). None of the tested strains produced detectable amounts of pyruvate, lactate, formate or ethanol.

Overall, these results indicate that the simultaneous deletion of *mdh* and *mqo* is a major factor towards decreased DHB formation, and that derivatives of this background may not be able to overproduce malate without secreting high amounts of the by-product acetate. The sole exception occurred for a mutant additionally devoid of the *poxB* gene, which produced DHB at levels comparable to those of the wild-type host strain, but no detectable amounts of malate or acetate.

### 3.3 The presence of the functional soluble malate dehydrogenase restores DHB and malate production in malate-optimized strains

**FIGURE 4:**
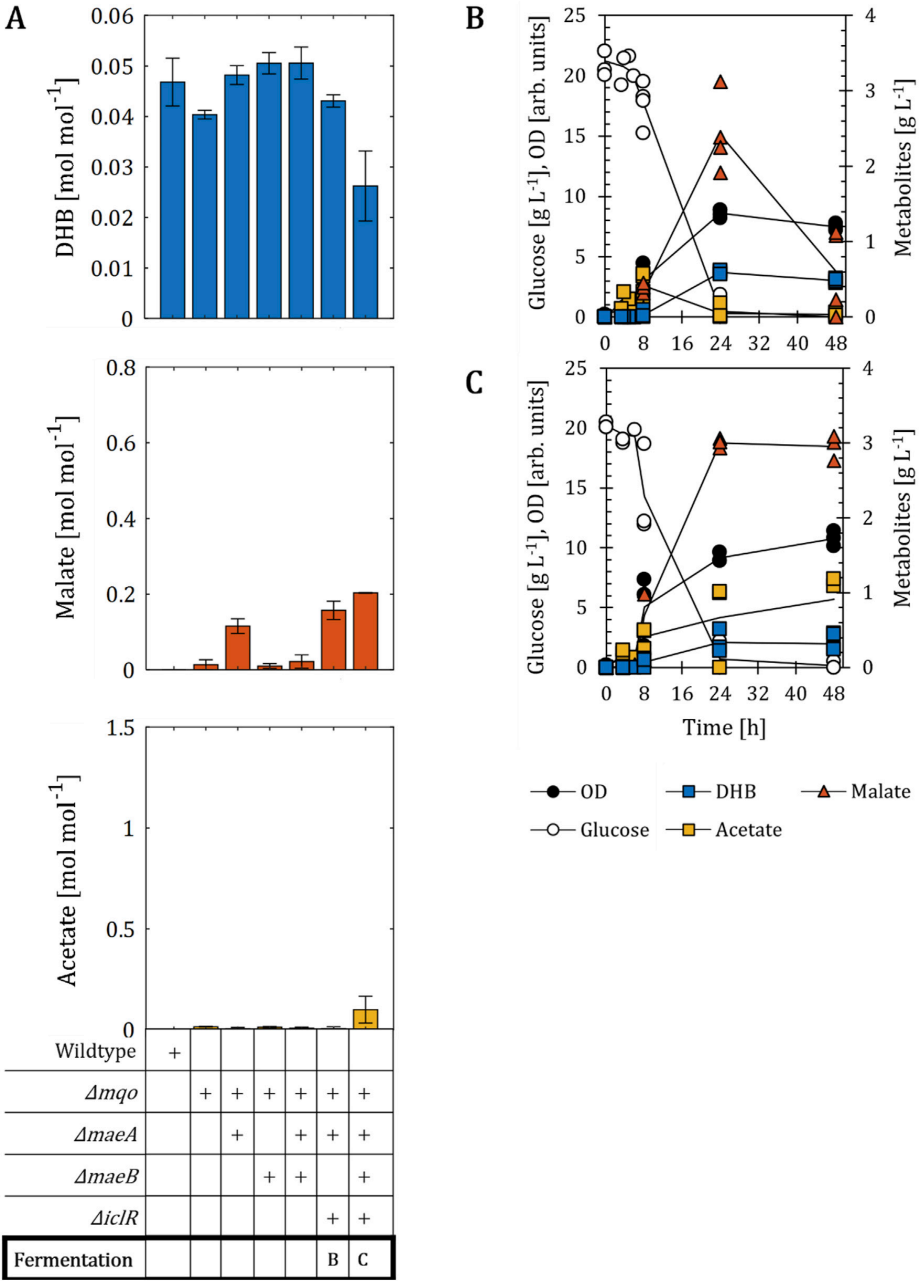
DHB production for *E. coli* mutants devoid of one malate dehydrogenase. **(A)** DHB, malate and acetate yields for *E. coli* mutants expressing the plasmid pZA33-DHBop-ppc_K620S_ after 24 h (48 h for wildtype and ∆*mqo*) of cultivation. Deletion (*∆*) of target genes is marked with a (+) sign. Fermentation profile of strains *Δmqo ΔmaeA ΔiclR* **(B)** and *Δmqo ΔmaeA ΔmaeB ΔiclR* **(C)**. All strains are derived from *E. coli* MG1655. Cell cultivation was performed in 250 mL baffled shake flasks (37°C, 220 rpm) with 25 mL M9 mineral medium. IPTG (1 mM) was added at mid-exponential phase to induce the expression of pathway genes. In **(A)** results correspond to the mean of at least two biological replicates is shown and error bars represent the standard deviation of the mean. In **(B,C)** individual data points are shown as colored dots. Lines are drawn to the mean values (see Supplementary Table S1).

Having shown that the deletion of all malate dehydrogenases impairs DHB production, we next decided to create an alternative setting devoid of only one malate dehydrogenase enzyme. We selected the single mutant *Δmqo* as the basis for the sequential deletion of *maeA*, *maeB* and *iclR* genes (Figure 4). We found the constructed host strains to nearly abolish the formation of acetate, thereby enabling us to infer the sole impact of intracellularly overproduced malate on the DHB synthetic pathway. We compared DHB, malate and acetate yields obtained after 24 h of cultivation, because malate was re-consumed between 24 and 48 h in strain backgrounds with intact *maeB* gene (data not shown). First, we observed that the simultaneous deletion of *maeB* and *mqo* genes had no significant impact on DHB (0.05 mol mol^-1^), malate (0.01 mol mol^-1^) or acetate (0.01 mol mol^-1^) yields compared to those obtained with the *Δmqo* mutant. On the other hand, the inactivation of the *maeA* gene resulted in an increase in malate production to a yield of 0.12 mol mol^-1^. This could also be observed in strain backgrounds in which the *iclR* gene is additionally deleted (0.16 mol mol^-1^). But in contrast to the strain *Δmqo ΔmaeA ΔmaeB ΔiclR*, malate was found to be re-assimilated for experiments with the strain *Δmqo ΔmaeA ΔiclR* from 2.4 g L^-1^ to 0.6 g L^-1^ between 24 and 48 h of cultivation (Figure 4B). DHB concentrations remained almost constant between 24 and 48 h (0.6 and 0.5 g L^-1^, respectively). With the additional deletion of *maeB* the malate concentration remained stable at 2.9 g L^-1^ (0.20 mol mol^-1^) even after glucose depletion (Figure 4C). Therefore, it is surprising that the strain *Δmqo ΔmaeA ΔmaeB* only produced trace amounts of malate (0.3 g L^-1^, Supplementary Table S1). Because acetate production is lower compared to that of strains devoid of both malate dehydrogenases, the deletion of *arcA*, which is commonly used to prevent acetate over-flow (Liu et al., 2016), was not considered in this setting. In regard to DHB production, it is of note that the best malate producer exhibited the lowest DHB production with a yield of 0.026 mol mol^-1^ (Figure 4A).

We conclude that the accumulation of high amounts of acetate impairs the production of DHB. However, the fact that malate still accumulated and no improved DHB formation was observed indicates the potential presence of metabolic inefficiencies downstream malate.

### 3.4 Inactivation of the NAD-dependent succinate semialdehyde dehydrogenase increases DHB production

In previous sections, we observed that an increased malate supply does not translate into improved DHB yields. While these findings could in principle be explained by a potential inhibition exerted by malate on the first step of the synthetic pathway, Walther et al. (2017) previously confirmed that the employed malate kinase enzyme is not inhibited at the range of malate concentrations disclosed in this work. Instead, our observations could be due to presence of an unwanted reaction in the endogenous metabolism of *E. coli* which could interconvert the DHB pathway intermediate MalSA to malate (Figure 1). Crucially, the chemical structure of the intermediate MalSA is similar to that of SucSA, a natural metabolite which takes part in the *γ*-aminobutyric acid (GABA)-shunt (Figure 1). Thus, we focused on the analysis of SucSA dehydrogenases which could potentially oxidize malate semialdehyde to yield malate. In *E. coli*, NAD(P)-dependent SucSA dehydrogenase activity is encoded by the genes *sad* and *gabD*.

**FIGURE 5:**
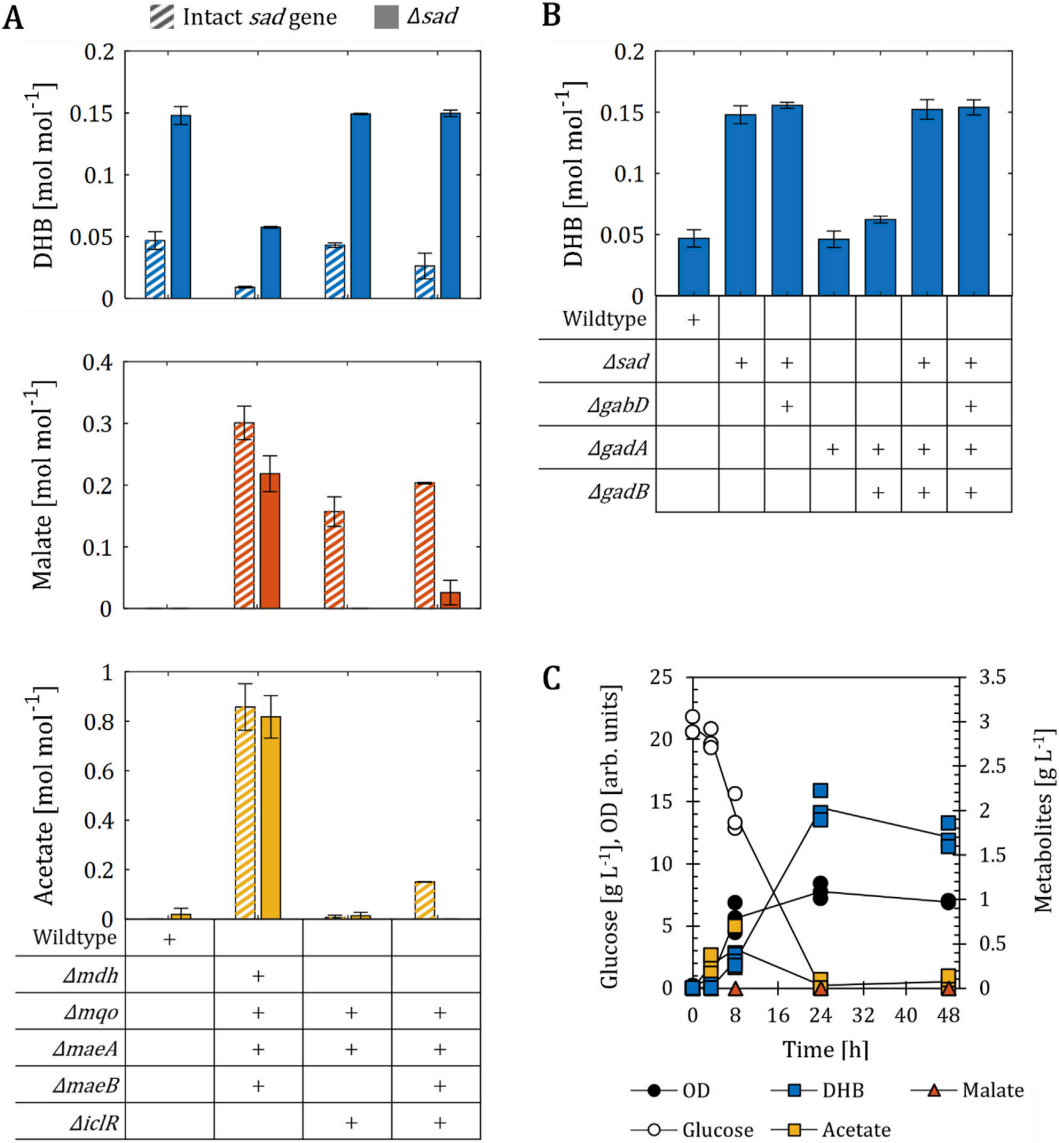
DHB production for *E. coli* mutants devoid of enzyme(s) implicated in the *γ*-aminobutyric acid (GABA)-shunt. **(A)** Influence of the deletion of NAD-dependent succinate semialdehyde dehydrogenase (*sad*) on DHB, malate and acetate yields after 24 h (48 h for wildtype, *Δmdh Δmqo ΔmaeA ΔmaeB* and *Δmdh Δmqo ΔmaeA ΔmaeB Δsad*) of cell cultivation of various strain backgrounds bearing the plasmid pZA33-DHBop-ppc_K620S_. Solid and stripped bars correspond to strains with and without *sad* gene, respectively. **(B)** Influence of the deletion of genes encoding enzyme activities implicated in the GABA shunt on DHB production. In **(A,B)** gene deletion (*∆*) is marked with a (+) sign. The mean of at least two biological replicates is shown and error bars represent the standard deviation of the mean. **(C)** Fermentation profile of *Δsad*. Individual data points are shown as colored dots. Lines are drawn to the mean values (see Supplementary Table S1). All strains are derived from *E. coli* MG1655. Cell cultivation was performed in 250 mL baffled shake flasks (37°C, 220 rpm) with 25 mL M9 mineral medium. IPTG (1 mM) was added at mid-exponential phase to induce the expression of pathway genes.

To test our hypothesis, we first deleted the *sad* gene from the wildtype host and transformed it with the plasmid pZA33-DHBop-ppc_K620S_ to investigate DHB production from glucose (Figure 5A). The sole knock-out of *sad* increased DHB production by 3-fold compared to the wildtype host (0.05–0.15 mol mol^-1^, respectively). But during the cultivation the re-assimilation of up to 0.4 g L^-1^ DHB could be observed between 24 and 48 h of cultivation after glucose depletion (Figure 5C). Therefore, DHB, malate and acetate yields after 24 h of cultivation were compared for the *sad* mutants, with the exception of the strain background *Δmdh Δmqo ΔmaeA ΔmaeB Δsad*. This strain was used to investigate the influence of *Δsad* in backgrounds which overproduce malate. The inactivation of Sad in this setting resulted in a restored DHB production to a yield of 0.06 mol mol^-1^ (Supplementary Table S2) and a small reduction of malate from 0.30 to 0.22 mol mol^-1^ compared to the predecessor strain *Δmdh Δmqo ΔmaeA ΔmaeB*. In this case we displayed the yields after 48 h of cultivation because both strains only consumed 13.9 g L^-1^ glucose at the end of the cultivation (Supplementary Table S1) with no observable DHB re-consumption (data not shown). But even though the quintuple mutant *Δmdh Δmqo ΔmaeA ΔmaeB Δsad* produced 6-times more DHB than its predecessor strain, the single *Δsad* mutant largely outperformed both strains. In an attempt to further increase DHB production, we additionally deleted the *sad* gene from host strains devoid of one sole malate dehydrogenase (*Δmqo ΔmaeA ΔiclR* and *Δmqo ΔmaeA ΔmaeB ΔiclR*) with the aim to redirect the carbon flux from malate back to DHB. In the strains *Δmqo ΔmaeA ΔiclR Δsad* and *Δmqo ΔmaeA ΔmaeB ΔiclR Δsad* the excretion of malate could be successfully prevented, but the DHB yield could not be increased above 0.15 mol mol^-1^ (Figure 5A). To observe the influence of the NADP-dependent SucSA dehydrogenase GabD, the deletion of *gabD* was introduced into the *Δsad* strain. However, the performance of the strains *Δsad* and *Δsad ΔgabD* was very similar (Figure 5B). Because Sad is implicated in the GABA-shunt, we further deleted genes coding for glutamate decarboxylases enzymes GadA and GadB, which decarboxylate L-glutamate to GABA (Smith et al., 1992) (see Figure 1). The additional deletion of *gadA* and *gadB* did not increase DHB yields (Figure 5B).

These results therefore confirm that the positive effect of the *sad* deletion was due to the removal of a previously unknown malate futile cycle, and not due to the interruption of the GABA shunt. We therefore retained the deletion of the *sad* gene as a minimal metabolic requirement for improved operation of the DHB pathway.

### 3.5 Chromosomal expression of the PEP carboxylase *ppc*_*K620S*_ increases DHB production

Having shown the requirements for DHB production, we attempted to integrate the MalP pathway first into the chromosome of the wildtype background. However, the integration of all the required pathway activities resulted in no detectable DHB production (data not shown), presumably because of the low catalytic efficiency of the enzymes linking malate to DHB (Walther et al., 2017). Thus, we turned our attention to the chromosomal integration of the gene *ppc*_*K620S*_, while keeping the other three remaining activities expressed from the medium-copy plasmid pZA33-DHBop. Supporting our rationale, we found that the removal of the *ppc*_*K620S*_ gene from the DHB production vector reduced plasmid loss with DHB producer strains (30%) by 2-fold after 24 h of cell cultivation when compared to strains bearing the pZA33-DHBop-ppc_K620S_ vector (plasmid loss, 59%) (Supplementary Figure S1). The increase of plasmid stability was presumably attained through the decrease in plasmid size (Cheah et al., 1987; Smith and Bidochka, 1998; Kreiss, 1999; Ertl and Thomsen, 2003; Friehs, 2004).

**FIGURE 6:**
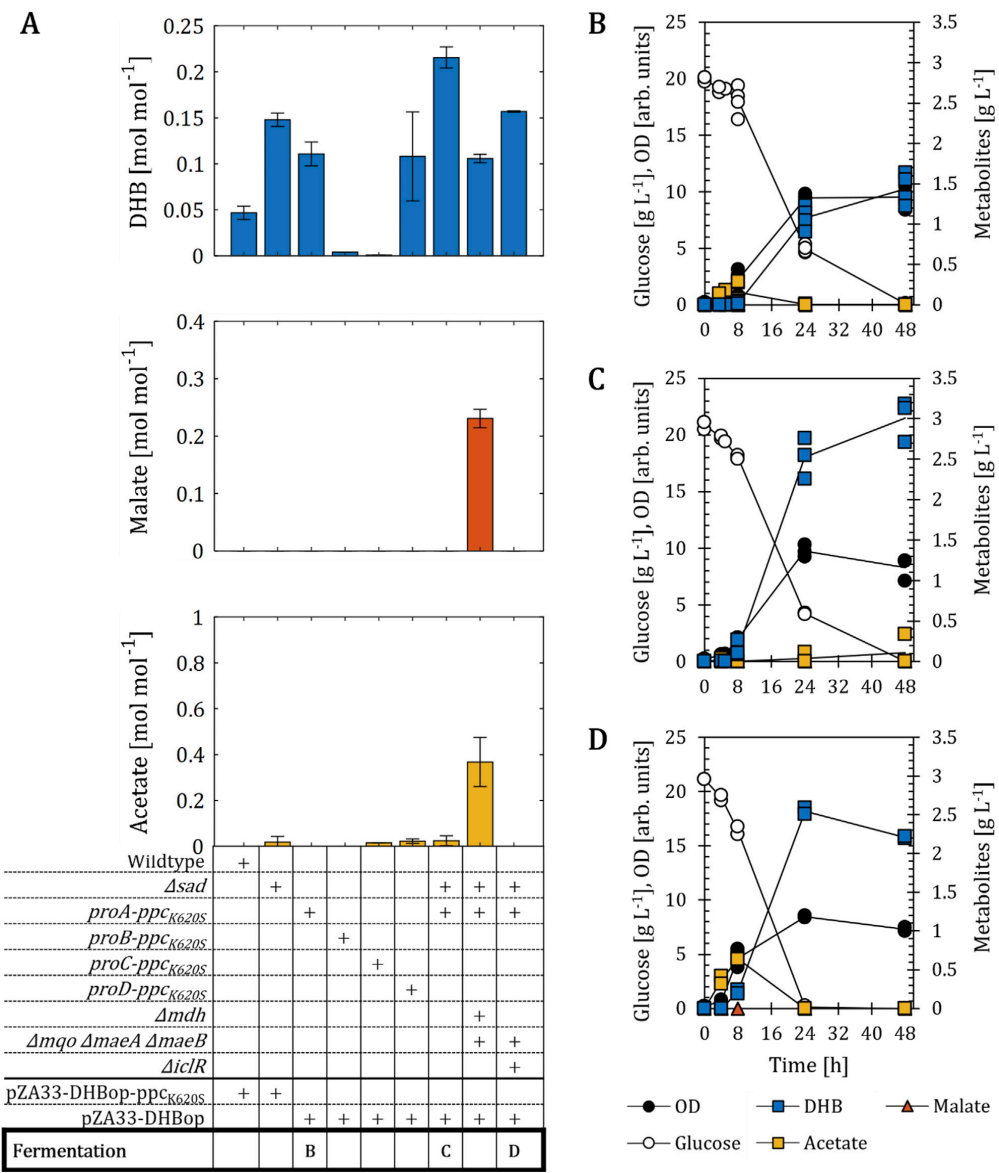
DHB production for *E. coli* mutants expressing chromosomally PEP carboxylase Ppc_K620S_. **(A)** DHB, malate and acetate yields after 48 h (24 h for *∆sad*) of cell cultivation. Strains constitutively expressing *ppc*_*K620S*_ from the chromosome are identified with respective synthetic promoter (proA-D) and contain the pZA33-DHBop plasmid, which contains all pathway activities with exception of PEP carboxylase. All remaining strains were transformed with the plasmid pZA33-DHBop-ppc_K620S_. Gene deletions (*∆*) introduced in target strains are marked with a (+) sign. Fermentation profile of *proA-ppc*_*K620S*_ **(B)**, *∆sad proA-ppc*_*K620S*_ **(C)** and *Δmdh Δmqo ΔmaeA ΔmaeB Δsad proA-ppc*_*K620S*_ **(D)** expressing the plasmid pZA33-DHBop. All cell cultivation was carried out in M9 mineral medium. IPTG (1 mM) was added at mid-exponential phase to induce the expression of pathway genes. In **(A)** the mean of at least two biological replicates is shown and error bars represent the standard deviation of the mean. In **(B–D)** individual data points are shown as colored dots. Lines are drawn to the mean values (see Supplementary Table S1).

To assess the effect of the chromosomal expression of PEP carboxylase, we replaced in the chromosome of *E. coli* the native *ppc* locus by *ppc*_*K620S*_ gene preceded by a constitutive promoter. In total, we investigated in wildtype cells bearing the plasmid pZA33-DHBop the expression of Ppc_K620S_ under control of four distinct promoters displaying variable transcriptional strengths (proA < proB < proC < proD) (Davis et al., 2011). The *proA-ppc*_*K620S*_ strain expressing the pZA33-DHBop plasmid showed 2.4-fold higher DHB production compared to the wildtype strain expressing the pZA33-DHBop-ppc_K620S_ plasmid (Figure 6A). Nonetheless, this strain still produced less DHB compared to the *sad* knock-out strain (0.11 and 0.15 mol mol^-1^, respectively). The use of the medium strength promoters proB and proC driving expression of the PEP carboxylase mutant led to the collapse of DHB production. The use of the strongest promoter proD did not result in further improved DHB yields. Even though the DHB production with this strain was comparable to that of *proA-ppc*_*K620S*_ mutant, the use of the promoter proA might be more beneficial as assessed by the higher growth rates (0.33 h^-1^ for *proA-ppc*_*K620S*_, and 0.2 h^-1^ for *proD-ppc*_*K620S*_ *;* Supplementary Table S1). Furthermore, the lower growth rate of the *proD-ppc*_*K620S*_ strain indicates a higher metabolic burden that was imposed on the cells due to higher expression levels of the *ppc*_*K620S*_ gene, which again resulted in a high variability in DHB production. For further strain engineering we therefore decided to combine the chromosomal expression of *ppc*_*K620S*_ under control of the proA promoter with the *sad* knock-out. The new mutant produced DHB at a yield of 0.22 mol mol^-1^, which corresponds to 4.4- and 1.5-fold increase compared to those achieved with the wildtype and *Δsad* strains, respectively. The integration of the *proA-ppc*_*K620S*_ into the background *Δmdh Δmqo ΔmaeA ΔmaeB Δsad* increased the DHB production from initially 0.07–0.11 mol mol^-1^, with a decrease in acetate formation from 0.9 to 0.4 mol mol^-1^ but no change in malate production compared to the parental strain expressing the wildtype *ppc* gene. This indicates that the *ppc*_*K620S*_ expression from the plasmid pZA33-DHBop-ppc_K620S_ was possibly too low for efficient conversion of glucose to DHB in the *Δmdh Δmqo* knock-out strains. The incorporation of the *proA-ppc*_*K620S*_ modification into the strain *Δmqo ΔmaeA ΔmaeB ΔiclR Δsad* did not increase DHB production compared to our best producer strain (Figures 6 A,D). The fermentation profiles of *proA-ppc*_*K620S*_ and *Δsad proA-ppc*_*K620S*_ host strains differ at the level of DHB and acetate (Figures 6B,C).

In the *proA-ppc*_*K620S*_ mutant, acetate was formed during the first 8 h of cultivation, while no DHB was detected. Acetate was subsequently re-consumed. This is a behavior which was already observed for the wildtype and *Δsad* backgrounds. On the other hand, the *Δsad proA-ppc*_*K620S*_ mutant showed no production of acetate in the first 8 h. Only at the end of cell cultivation small amounts of acetate were detected (0.1 g L^-1^). The concentrations of DHB produced by the two strains at 48 h were distinct, with 1.4 g L^-1^ for the *proA-ppc*_*K620S*_ host strain and 3.0 g L^-1^ for the *Δsad proA-ppc*_*K620S*_ background. Because the deletion of *sad* could also increase the intracellular pool of SucSA, we performed additional LC/MS analysis for the best producer strain to confirm the production of DHB and not 4-hydoxybutyrate, which can be naturally derived through endogenous 4-hydroxybutyric dehydrogenases (Saito et al., 2009).

Overall, with the substitution of the natural *ppc* gene with the mutant variant *ppc*_*K620S*_ under the control of the weak proA promoter, it was possible to use the more stable pZA33-DHBop production plasmid. In combination with the *Δsad* we achieved a DHB yield of 0.22 mol mol^-1^, that is 17% of the maximal theoretical DHB yield on glucose under aerobic conditions.

### 3.6 Discussion

In this study we investigated the metabolic requirements to improve DHB production via the MalP pathway in *E. coli*. We first investigated the effect of an increased supply of the precursor malate by incorporating the synthetic DHB pathway into a previously established aerobic malate producer strain (Trichez et al., 2018). This strain harbored deletions for all malate dehydrogenases and malic enzymes, as well as the transcriptional regulators IclR and ArcA. But even though an increased supply of malate should, in theory, have been beneficial for the biosynthesis of products derived from malate, DHB production surprisingly collapsed. Instead, high amounts of acetate were produced and excess malate was excreted from cells. We hypothesize that the simultaneous deletion of both malate dehydrogenases (whose reaction favors malate oxidation under aerobic conditions) creates an imbalance between oxaloacetate and acetyl-CoA in the cell, which ultimately results in acetate secretion and hampers DHB production. As low DHB production was also observed with strains overproducing malate but negligible amounts of acetate, we hypothesized that metabolic inefficiencies of the pathway occur downstream malate. Indeed, we found the NAD-dependent SucSA dehydrogenase Sad (Fuhrer et al., 2007), whose natural substrate is structurally similar to MalSA, to be implicated in a previously unknown malate futile cycle, as the single knock-out of *sad* increased the DHB yield by 3-fold compared to the wildtype host. Because Sad was already reported to be a promiscuous enzyme, which is able to convert the C3-compound malonic semialdehyde to malonic acid (Song et al., 2016), we suggest that this enzyme was responsible for the unwanted reaction of MalSA oxidation into malate. The production of DHB requires, alongside the direct pathway enzymes, the overexpression of the malate-insensitive *ppc*_*K620S*_ gene to prevent the allosteric inhibition of the enzyme by malate (Yano and Izui, 1997; Walther et al., 2017). But previous studies show that the production of TCA-derived value-added compounds requires carefully modulated expression of the anaplerotic enzyme (Lee et al., 2007; Tan et al., 2013; Trichez et al., 2018). Therefore, we decided to chromosomally integrate the mutant *ppc*_*K620S*_ and replace the natural promoter with the weak, constitutive proA promoter (Davis et al., 2011). In combination with the inactivation of Sad, we could raise the DHB yield to 0.22 mol mol^-1^.

But even though DHB production could be increased by more than 4-fold compared to the wildtype host, the capacity of the MalP-dependent pathway is not fully exploited yet. In particular, DHB re-consumption observed for *Δsad* mutants can be prevented by the additional deletion of either endogenous DHB-importer systems (GlcA, LldP) or membrane-bound DHB dehydrogenase (LldD) (Frazão et al., 2019). In addition, our synthetic pathway could benefit from an increased supply of the NADPH co-factor, as MalSA dehydrogenase and reductase enzymes are NADPH-dependent enzymes (Walther et al., 2017). This could be achieved by increasing flux through pentose-P pathway by deleting glucose-6P isomerase (Pgi) (Kabir and Shimizu, 2003; Chemler et al., 2010) or phosphofructokinase (PfkA) (Wang et al., 2013; Siedler et al., 2014) activities, or by increasing activity of endogenous membrane-bound transhydrogenase PntAB (NADH + NADP - > NAD + NADPH + H^+^) (Rathnasingh et al., 2012; Shi et al., 2013; Ihle et al., 2025). The additional observation that only a fraction of the carbon (44% for our best DHB producer strain) could be recovered in the form of biomass and quantified metabolites indicates that a significant portion of the substrate and/or pathway intermediates was deviated from the pathway. Therefore, it is important to close the carbon balance by discovering and blocking the main carbon sinks, which could range from TCA derived fatty, amino and organic acids (Schulz and Dickschat, 2007; Audrain et al., 2015) to gaseous fatty acids derivatives and terpenoids (Carneiro et al., 2011). Additionally, it may be required to chromosomally integrate all pathway activities to achieve industrially relevant DHB titers. While in this study we could not generate strains producing DHB with the entire DHB operon chromosomally integrated, we consider that in part, this was due to the low catalytic efficiency of the employed malate semialdehyde dehydrogenase enzyme. Further enzyme engineering of rate-limiting pathway activities and careful optimization of 5′-UTR regions (promoter, and ribosome binding site) driving expression of each of the pathway enzymes may enable stable DHB production with chromosomally integrated strains. If these bottle-necks are addressed, the following step would be the up-scaling of the process, in which the attention should be focused on the toxicity that higher quantities of DHB as an acid will impose on cells and adequate aeration of the fermentation.

In summary, we showed an improvement of the MalP-dependent DHB production pathway from 0.05 to 0.22 mol mol^-1^, which equals 17% of the maximal DHB yield. Even though we did not yet reach industrially relevant DHB titers, we here showed that pathway performance can be increased with a minimal strain engineering approach. In combination with further strain engineering and identification of carbon wasting pathways, we expect that further increased DHB production levels can be achieved.

## Data Availability

The original contributions presented in the study are included in the article/Supplementary Material, further inquiries can be directed to the corresponding author.
