# Supplementary material for: Deletion of succinic semialdehyde dehydrogenase sad and chromosomal expression of phosphoenolpyruvate carboxylase as metabolic requirements for improved production of 2,4-dihydroxybutyric acid via malyl-P pathway using E. coli
Source: Front Bioeng Biotechnol. 2025 May 12;13:1589489. doi: 10.3389/fbioe.2025.1589489 (PMC12104201; doi:10.3389/fbioe.2025.1589489)
Supplement: Supplementary file 1 [file DataSheet1.docx]

Supplementary Material

**Deletion of succinic semialdehyde dehydrogenase Sad and chromosomal expression of phosphoenolpyruvate carboxylase as metabolic requirements for improved production of 2,4-dihydroxybutyric acid via malyl-P pathway using E. coli**

**T.A. Stefanie Nguyen^1^, Ceren Alkim^2^, Nadine Ihle^1^, Thomas Walther^1*^, Cláudio J.R. Frazão^1^**

^1^Chair of Bioprocess Engineering, Institute of Natural Materials Technology, TU Dresden, Bergstraße 120, 01062 Dresden, Germany

^2^Toulouse Biotechnology Institute, UMR INSA-CNRS5504 and UMR INSA-INRAE 792; 135 avenue de Rangueil, 31077 Toulouse, France

***Correspondence:**

Prof. Thomas Walther

Email: thomas_walther@tu-dresden.de

**Contents summary:**

Table S1 Concentration of consumed glucose (Glc), production of DHB, malate, acetate and biomass shown as OD_600_ after 24 or 48 hours of cultivation, and exponential growth rate (µ_exp_) of used strains. Not detected metabolites are indicated as n.d.

Table S2 Corresponding yield (Y) of DHB, malate (Mal), acetate (Ac), fumarate (Fum) and succinate (Suc) as well as the Sum of carbon (without consideration of CO_2_) after 24 or 48 hours of cultivation. Not detected metabolites are indicated as n.d.

Figure S1 DHB production and plasmid loss after 24 hours of cultivation in M9 mineral media with 20 g/L glucose. The used plasmids were transformed into wildtype E. coli MG1655 cells.

Table S3 Primers used in this study.

Table S4 Concentration of consumed glucose (Glc), production of DHB, malate, acetate and biomass shown as OD_600_ after 24 or 48 hours of cultivation, and exponential growth rate (µ_exp_) of used strains. Not detected metabolites are indicated as n.d.

| **Strain** | **Plasmid** | **Time** | **Consumed Glc [g L^-1^]** | **DHB**  **[g L^-1^]** | **Malate**  **[g L^-1^]** | **Acetate**  **[g L^-1^]** | **OD_600_**  **[-]** | **µ_exp_**  **[h^-1^]** |
| --- | --- | --- | --- | --- | --- | --- | --- | --- |
| Wildtype | pZA33-DHBop-ppc_K620S_ | 48h | 17.1 ± 1.6 | 0.6 ± 0.1 | n.d. | n.d. | 7.1 ± 0.4 | 0.39 ± 0.03 |
| *Δmdh Δmqo ΔmaeA ΔmaeB ΔiclR ΔarcA* (Mal-op) | pACT3-ppc_K620S_ | 48h | 14.5 ± 0.3 | n.d. | 5.3 ± 0.1 | 2.5 ± 0.5 | 8.0 ± 0.4 | 0.14 ± 0.03 |
| Mal-op | pACT3-ppc_K620S_-gltA* | 48h | 13.0 ± 3.5 | n.d. | 5.6 ± 2.2 | 0.8 ± 0.5 | 8.4 ± 0.5 | 0.12 ± 0.00 |
| Mal-op | pACT3-ppc_K620S_;  pZS15-gltA_R164L_ | 48h | 14.3 ± 0.8 | n.d. | 5.5 ± 0.4 | 2.1 ± 0.3 | 8.0 ± 0.2 | 0.13 ± 0.00 |
| Mal-op | pZA33-DHBop-ppc_K620S_ | 48h | 12.5 ± 1.0 | 0.1 ± 0.0 | 3.6 ± 0.3 | 3.8 ± 0.1 | 6.1 ± 0.5 | 0.17 ± 0.05 |
| Mal-op | pZA33-DHBop-ppc_K620S_;  pZS15- gltA_R164L_ | 48h | 13.6 ± 0.4 | 0.1 ± 0.0 | 3.4 ± 0.3 | 3.5 ± 0.5 | 4.7 ± 0.2 | 0.16 ± 0.03 |
| *Δmdh* | pZA33-DHBop-ppc_K620S_ | 48h | 19.8 ± 0.3 | 0.4 ± 0.1 | 0.2 ± 0.2 | 1.6 ± 0.7 | 7.1 ± 1.1 | 0.38 ± 0.01 |
| *Δmqo* | pZA33-DHBop-ppc_K620S_ | 48h | 19.8 ± 0.1 | 0.5 ± 0.0 | 0.2 ± 0.2 | 0.1 ± 0.0 | 9.3 ± 0.3 | 0.37 ± 0.01 |
| *Δmdh Δmqo* | pZA33-DHBop-ppc_K620S_ | 48h | 18.0 ± 0.9 | 0.1 ± 0.0 | 1.7 ± 0.1 | 7.3 ± 0.2 | 6.8 ± 1.4 | 0.36 ± 0.00 |
| *Δmdh Δmqo ΔmaeA ΔmaeB* | pZA33-DHBop-ppc_K620S_ | 48h | 13.9 ± 0.0 | 0.1 ± 0.0 | 3.6 ± 0.2 | 4 ± 0.4 | 6.4 ± 0.6 | 0.27 ± 0.01 |
| *Δmdh Δmqo ΔmaeA ΔmaeB ΔiclR* | pZA33-DHBop-ppc_K620S_ | 48h | 13.2 ± 1.0 | 0.1 ± 0.0 | 3.0 ± 0.2 | 4.6 ± 0.6 | 5.5 ± 0.5 | 0.27 ± 0.00 |
| Δmdh Δmqo ΔmaeA ΔmaeB ΔackA-pta | pZA33-DHBop-ppc_K620S_ | 48h | 12.0 ± 0.8 | n.d. | 0.6 ± 0.3 | 4.3 ± 0.2 | 3.5 ± 0.4 | 0.16 ± 0.00 |
| *Δmdh Δmqo ΔmaeA ΔmaeB ΔarcA ΔackA-pta* | pZA33-DHBop-ppc_K620S_ | 48h | 14.2 ±0.4 | 0.1 ± 0.0 | 5.2 ± 0.1 | 2.8 ± 0.2 | 8.7 ± 0.1 | 0.21 ± 0.01 |
| *Δmdh Δmqo ΔmaeA ΔmaeB ΔpoxB* | pZA33-DHBop-ppc_K620S_ | 48h | 20.0 ± 0.7 | 0.6 ± 0.1 | 0.1 ± 0.1 | n.d. | 9.7 ± 2.7 | 0.36 ± 0.09 |
| *Δmqo ΔmaeA* | pZA33-DHBop-ppc_K620S_ | 24h | 19.9 ± 0.3 | 0.6 ± 0.0 | 1.7 ± 0.3 | n.d. | 9.3 ± 0.2 | 0.39 ± 0.03 |
| *Δmqo ΔmaeB* | pZA33-DHBop-ppc_K620S_ | 24h | 18.7 ± 1.1 | 0.7 ± 0.1 | 0.1 ± 0.1 | 0.1 ± 0.0 | 8.7 ± 0.6 | 0.4 ± 0.02 |
| *Δmqo ΔmaeA ΔmaeB* | pZA33-DHBop-ppc_K620S_ | 24h | 18.3 ± 1.2 | 0.6 ± 0.1 | 0.3 ± 0.2 | 0.1 ± 0.0 | 9.7 ± 1.8 | 0.35 ± 0.03 |
| *Δmqo ΔmaeA ΔiclR* | pZA33-DHBop-ppc_K620S_ | 24h | 20.7 ± 1.3 | 0.6 ± 0.0 | 2.4 ± 0.4 | 0.1 ± 0.1 | 8.0 ± 0.7 | 0.34 ± 0.04 |
| *Δmqo ΔmaeA ΔmaeB ΔiclR* | pZA33-DHBop-ppc_K620S_ | 24h | 19.5 ± 0.8 | 0.3 ± 0.1 | 2.9 ± 0.1 | 0.7 ± 0.4 | 10.8 ± 0.4 | 0.39 ± 0.07 |
| *Δsad* | pZA33-DHBop-ppc_K620S_ | 24h | 20.7 ± 0.5 | 2.0 ± 0.1 | n.d. | n.d. | 7.6 ± 0.0 | 0.42 ± 0.00 |
| *Δmdh Δmqo ΔmaeA ΔmaeB Δsad* | pZA33-DHBop-ppc_K620S_ | 48h | 13.9 ± 1.5 | 0.5 ± 0.1 | 2.2 ± 0.1 | 3.7 ± 0.0 | 6.8 ± 0.0 | 0.31 ± 0.00 |
| *Δmqo ΔiclR ΔmaeA ∆sad* | pZA33-DHBop-ppc_K620S_ | 24h | 20.9 ± 0.2 | 2.1 ± 0.0 | n.d. | n.d. | 9.3 ± 0.0 | 0.36 ± 0.00 |
| *Δmqo ΔmaeA ΔmaeB ΔiclR ∆sad* | pZA33-DHBop-ppc_K620S_ | 24h | 20.9 ± 0.6 | 2.1 ± 0.1 | n.d. | n.d. | 9.4 ± 0.0 | 0.41 ± 0.00 |
| *Δsad ΔgabD* | pZA33-DHBop-ppc_K620S_ | 24h | 20.1 ± 0.1 | 2.0 ± 0.1 | n.d. | n.d. | 7.0 ± 0.2 | 0.42 ± 0.01 |
| *ΔgadA* | pZA33-DHBop-ppc_K620S_ | 24h | 20.9 ± 0.6 | 0.6 ± 0.1 | n.d. | n.d. | 8.5 ± 0.5 | 0.42 ± 0.01 |
| *ΔgadA ΔgadB* | pZA33-DHBop-ppc_K620S_ | 24h | 20.9 ± 0.6 | 0.9 ± 0.0 | n.d. | n.d. | 8.4 ± 0.4 | 0.41 ± 0.00 |
| *ΔgadA ΔgadB Δsad* | pZA33-DHBop-ppc_K620S_ | 24h | 20.9 ± 0.6 | 2.1 ± 0.2 | n.d. | n.d. | 8.0 ± 0.5 | 0.42 ± 0.01 |
| *ΔgadA ΔgadB Δsad ΔgabD* | pZA33-DHBop-ppc_K620S_ | 24h | 20.9 ± 0.6 | 2.1 ± 0.1 | n.d. | n.d. | 8.1 ± 0.1 | 0.41 ± 0.01 |
| *proA- ppc_K620S_* (kanR) | pZA33-DHBop | 48h | 19.9 ± 0.2 | 1.5 ± 0.2 | n.d. | n.d. | 9.9 ± 0.3 | 0.33 ± 0.02 |
| *proB- ppc_K620S_* (kanR) | pZA33-DHBop | 48h | 15.7 ± 0.0 | n.d. | n.d. | n.d. | 10.1 ± 0.0 | 0.01 ± 0.00 |
| *proC- ppc_K620S_* (kanR) | pZA33-DHBop | 48h | 12.2 ± 0.0 | n.d. | n.d. | n.d. | 9.5 ± 0.0 | 0.04 ± 0.00 |
| *proD- ppc_K620S_* (kanR) | pZA33-DHBop | 48h | 18.8 ± 1.0 | 1.4 ± 0.6 | n.d. | n.d. | 10.0 ± 0.4 | 0.20 ± 0.05 |
| *Δsad proA-ppc_K620S_* (kanR) | pZA33-DHBop | 48h | 16.7 ± 0.3 | 3.0 ± 0.2 | n.d. | n.d. | 8.3 ± 0.8 | 0.28 ± 0.01 |
| *Δmdh Δmqo ΔmaeA ΔmaeB Δsad proA-ppc_K620S_* (kanR) | pZA33-DHBop | 48h | 17.8 ± 0.1 | 1.2 ± 0.1 | 3.0 ± 0.4 | 2.1 ± 0.5 | 7.2 ± 0.9 | 0.15 ± 0.01 |
| *Δmqo ΔmaeAB ΔiclR Δsad proA-ppc_K620S_* (kanR) | pZA33-DHBop | 48h | 21.0 ± 0.1 | 2.2 ± 0.0 | n.d. | n.d. | 7.3 ± 0.2 | 0.38 ± 0.02 |

Table S5 Corresponding yield (Y) of DHB, malate (Mal), acetate (Ac), fumarate (Fum) and succinate (Suc) as well as the Sum of carbon (without consideration of CO_2_) after 24 or 48 hours of cultivation. Not detected metabolites are indicated as n.d.

| **Strain** | **Plasmid** | **Time** | **Y_DHB**  **[mol mol^-1^]** | **Y_Mal**  **[mol mol^-1^]** | **Y_Ac**  **[mol mol^-1^]** | **Y_Fum**  **[mol mol^-1^]** | **Y_Suc**  **[mol mol^-1^]** | **Sum of carbon [%]**  **(without CO_2_)** |
| --- | --- | --- | --- | --- | --- | --- | --- | --- |
| Wildtype | pZA33-DHBop-ppc_K620S_ | 48h | 0.05 ± 0.01 | n.d. | n.d. | n.d. | n.d. | 23.0 ± 2.3 |
| *Δmdh Δmqo ΔmaeA ΔmaeB ΔiclR ΔarcA* (Mal-op) | pACT3-ppc_K620S_ | 48h | n.d. | 0.49 ± 0.01 | 0.52 ± 0.09 | 0.06 ± 0.01 | 0.01 ± 0.00 | 80.4 ± 6.7 |
| Mal-op | pACT3-ppc_K620S_-gltA* | 48h | n.d. | 0.55 ± 0.10 | 0.2 ± 0.09 | 0.12 ± 0.08 | 0.02 ± 0.02 | 84.7 ± 9.9 |
| Mal-op | pACT3-ppc_K620S_;  pZS15-gltA_R164L_ | 48h | n.d. | 0.52 ± 0.02 | 0.44 ± 0.07 | 0.05 ± 0.02 | 0.01 ± 0.01 | 71.4 ± 12.2 |
| Mal-op | pZA33-DHBop-ppc_K620S_ | 48h | 0.01 ± 0.00 | 0.39 ± 0.05 | 0.92 ± 0.08 | 0.09 ± 0.03 | 0.01 ± 0.01 | 85.7 ± 10.1 |
| Mal-op | pZA33-DHBop-ppc_K620S_;  pZS15- gltA_R164L_ | 48h | 0.01 ± 0.00 | 0.34 ± 0.04 | 0.78 ± 0.12 | 0.07 ± 0.01 | 0.01 ± 0.01 | 70.3 ± 9.1 |
| *Δmdh* | pZA33-DHBop-ppc_K620S_ | 48h | 0.03 ± 0.00 | 0.02 ± 0.02 | 0.25 ± 0.10 | n.d. | n.d. | 28.8 ± 4.2 |
| *Δmqo* | pZA33-DHBop-ppc_K620S_ | 48h | 0.04 ± 0.00 | 0.01 ± 0.01 | 0.02 ± 0.00 | n.d. | n.d. | 27.2 ± 1.8 |
| *Δmdh Δmqo* | pZA33-DHBop-ppc_K620S_ | 48h | 0.01 ± 0.00 | 0.13 ± 0.00 | 1.21 ± 0.03 | n.d. | 0.02 ± 0.00 | 68.4 ± 2.5 |
| *Δmdh Δmqo ΔmaeA ΔmaeB* | pZA33-DHBop-ppc_K620S_ | 48h | 0.01 ± 0.00 | 0.3 ± 0.03 | 0.86 ± 0.09 | 0.07 ± 0.02 | 0.02 ± 0.00 | 80.6 ± 1.0 |
| *Δmdh Δmqo ΔmaeA ΔmaeB ΔiclR* | pZA33-DHBop-ppc_K620S_ | 48h | 0.02 ± 0.00 | 0.31 ± 0.01 | 1.08 ± 0.25 | 0.08 ± 0.02 | 0.03 ± 0.01 | 83.1 ± 5.7 |
| Δmdh Δmqo ΔmaeA ΔmaeB ΔackA-pta | pZA33-DHBop-ppc_K620S_ | 48h | n.d. | 0.06 ± 0.03 | 1.08 ± 0.11 | 0.02 ± 0.00 | n.d. | 55.3 ± 1.0 |
| *Δmdh Δmqo ΔmaeA ΔmaeB ΔarcA ΔackA-pta* | pZA33-DHBop-ppc_K620S_ | 48h | 0.01 ± 0.00 | 0.48 ± 0.01 | 0.58 ± 0.04 | 0.07 ± 0.01 | 0.03 ± 0.01 | 82.0 ± 0.4 |
| *Δmdh Δmqo ΔmaeA ΔmaeB ΔpoxB* | pZA33-DHBop-ppc_K620S_ | 48h | 0.04 ± 0.01 | 0.01 ± 0.01 | n.d. | 0.01 ± 0.01 | 0.01 ± 0.01 | 28.5 ± 6.9 |
| *Δmqo ΔmaeA* | pZA33-DHBop-ppc_K620S_ | 24h | 0.05 ± 0.00 | 0.12 ± 0.02 | n.d. | 0.04 ± 0.00 | 0.02 ± 0.00 | 36.4 ± 2.1 |
| *Δmqo ΔmaeB* | pZA33-DHBop-ppc_K620S_ | 24h | 0.05 ± 0.00 | 0.01 ± 0.01 | 0.01 ± 0.00 | 0.02 ± 0.01 | 0.02 ± 0.02 | 30.4 ± 2.7 |
| *Δmqo ΔmaeA ΔmaeB* | pZA33-DHBop-ppc_K620S_ | 24h | 0.05 ± 0.00 | 0.02 ± 0.02 | 0.01 ± 0.01 | 0.01 ± 0.02 | 0.01 ± 0.01 | 32.1 ± 2.3 |
| *Δmqo ΔmaeA ΔiclR* | pZA33-DHBop-ppc_K620S_ | 24h | 0.04 ± 0.00 | 0.16 ± 0.02 | 0.01 ± 0.01 | 0.03 ± 0.01 | 0.01 ± 0.01 | 35.3 ± 2.0 |
| *Δmqo ΔmaeA ΔmaeB ΔiclR* | pZA33-DHBop-ppc_K620S_ | 24h | 0.03 ± 0.01 | 0.2 ± 0.00 | 0.10 ± 0,07 | 0.04 ± 0.00 | 0.02 ± 0.01 | 49.6 ± 0.3 |
| *Δsad* | pZA33-DHBop-ppc_K620S_ | 24h | 0.15 ± 0.01 | n.d. | 0.02 ± 0.02 | n.d. | n.d. | 28.9 ± 2.7 |
| *Δmdh Δmqo ΔmaeA ΔmaeB Δsad* | pZA33-DHBop-ppc_K620S_ | 48h | 0.06 ± 0.00 | 0.22 ± 0.03 | 0.82 ± 0.09 | n.d. | n.d. | 71.8 ± 8.6 |
| *Δmqo ΔiclR ΔmaeA ∆sad* | pZA33-DHBop-ppc_K620S_ | 24h | 0.15 ± 0.00 | n.d. | 0.01 ± 0.01 | n.d. | n.d. | 35.2 ± 1.0 |
| *Δmqo ΔmaeA ΔmaeB ΔiclR ∆sad* | pZA33-DHBop-ppc_K620S_ | 24h | 0.15 ± 0.00 | n.d. | n.d. | n.d. | n.d. | 35.1 ± 2.3 |
| *Δsad ΔgabD* | pZA33-DHBop-ppc_K620S_ | 24h | 0.16 ± 0.00 | n.d. | 0.01 ± 0.01 | n.d. | n.d. | 28.9 ± 0.9 |
| *ΔgadA* | pZA33-DHBop-ppc_K620S_ | 24h | 0.05 ± 0.01 | n.d. | n.d. | n.d. | n.d. | 24.3 ± 1.2 |
| *ΔgadA ΔgadB* | pZA33-DHBop-ppc_K620S_ | 24h | 0.06 ± 0.00 | n.d. | n.d. | n.d. | n.d. | 25.2 ± 1.5 |
| *ΔgadA ΔgadB Δsad* | pZA33-DHBop-ppc_K620S_ | 24h | 0.15 ± 0.01 | n.d. | n.d. | n.d. | n.d. | 30.4 ± 0.9 |
| *ΔgadA ΔgadB Δsad ΔgabD* | pZA33-DHBop-ppc_K620S_ | 24h | 0.15 ± 0.01 | n.d. | n.d. | n.d. | n.d. | 30.5 ± 0.2 |
| *proA- ppc_K620S_* (kanR) | pZA33-DHBop | 48h | 0.11 ± 0.01 | n.d. | n.d. | n.d. | n.d. | 31.9 ± 1.9 |
| *proB- ppc_K620S_* (kanR) | pZA33-DHBop | 48h | n.d. | n.d. | n.d. | n.d. | n.d. | 31.9 |
| *proC- ppc_K620S_* (kanR) | pZA33-DHBop | 48h | n.d. | n.d. | 0.02 | n.d. | n.d. | 39.1 |
| *proD- ppc_K620S_* (kanR) | pZA33-DHBop | 48h | 0.11 ± 0.05 | n.d. | 0.02 ± 0.01 | n.d. | n.d. | 33.5 ± 3.7 |
| *Δsad proA-ppc_K620S_* (kanR) | pZA33-DHBop | 48h | 0.22 ± 0.01 | n.d. | 0.02 ± 0.02 | n.d. | n.d. | 44.1 ± 1.6 |
| *Δmdh Δmqo ΔmaeA ΔmaeB Δsad proA-ppc_K620S_* (kanR) | pZA33-DHBop | 48h | 0.11 ± 0.00 | 0.23 ± 0.02 | 0.37 ± 0.11 | 0.02 ± 0.02 | n.d. | 56.9 ± 1.8 |
| *Δmqo ΔmaeAB ΔiclR Δsad proA-ppc_K620S_* (kanR) | pZA33-DHBop | 48h | 0.16 ± 0.00 | n.d. | n.d. | n.d. | 0.01 ± 0.01 | 30.5 ± 2.4 |


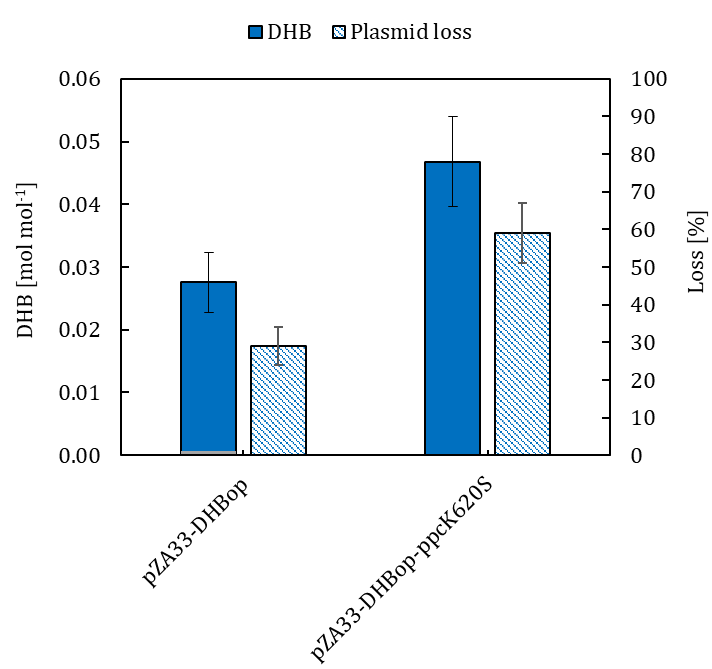


Figure S2 DHB production and plasmid loss after 24 hours of cultivation in M9 mineral media with 20 g L^-1^ glucose. The used plasmids were transformed into wildtype E. coli MG1655 cells.

Table S6 Primers used in this study.

| **Name** | **Sequence (5'-->3')** |
| --- | --- |
| *Cloning primer* | |
| XhoI-pTAC-gltA_fwd | TAAGCActcgagcggagcttatcgactgcac |
| HindIII-gltA_rev | TGCTTAaagcttTTAACGCTTGATATCGCTTTTA |
| *Knock-out verification primer* | |
| mdh_locus_f | GTTGGAATGTTGCGCTAATGC |
| mdh_locus_r | GTATTCAGGTCAACGATCTG |
| mqo_locus_f | actgctgccgtcaggtcaat |
| mqo_locus_r | tatcagcatacgccacatccg |
| maeA(sfcA)_locus_f | tagtaaataacccaaccggc |
| maeA(sfcA)_locus_r | tacgtaacgtcaacatgatg |
| maeB_locus_f | atggtattgctggattaagc |
| maeB_locus_r | agatcactaagaatggagag |
| iclR_locus_f | CGACCACCACGCAACATGA |
| iclR_locus_r | GGCGTCAATGCGATTAACAG |
| arcA_locus_f | gcgtagttttattgggtgtc |
| arcA_locus_r | ctgctcaactctgccgatag |
| ackA_locus_f | GCCTGAAGGCCTAAGTAGTAC |
| ackA_locus_r | CACGATAGTCGTAGTCTGATCG |
| poxB_locus_f | CGATGATATTCCTTTCATCGGGC |
| poxB_locus_r | CGTAAATCAATCATGGCATGTCC |
| sad_locus_f | tagtgagattcgacggcacg |
| sad_locus_r | cagatagcaatccccaatcg |
| gabD_locus_f | ttctgtttgtcaccaccccg |
| gabD_locus_r | atgcaggtgcccggtattga |
| gadA_locus_f | gccgtatatgcaggataaac |
| gadA_locus_r | cacagggaaattaatagtaagc |
| gadB_locus_f | aggagacacagaatgcgc |
| gadB_locus_r | gtaatgtgagctgcttagc |
| ppc_locus_f | caccatttttgctggcattaa |
| ppc_locus_r | TCCACGAAGTAAAACGGACC |
